# A combined gene expression tool for parallel histological prediction and gene fusion detection in non-small cell lung cancer

**DOI:** 10.1038/s41598-019-41585-4

**Published:** 2019-03-26

**Authors:** Anna Karlsson, Helena Cirenajwis, Kajsa Ericson-Lindquist, Hans Brunnström, Christel Reuterswärd, Mats Jönsson, Cristian Ortiz-Villalón, Aziz Hussein, Bengt Bergman, Anders Vikström, Nastaran Monsef, Eva Branden, Hirsh Koyi, Luigi de Petris, Patrick Micke, Annika Patthey, Annelie F. Behndig, Mikael Johansson, Maria Planck, Johan Staaf

**Affiliations:** 1 Division of Oncology and Pathology, Department of Clinical Sciences Lund, Lund University, Medicon Village, SE 22381 Lund, Sweden; 2 Division of Oncology and Pathology, Department of Clinical Sciences Lund, Lund University, SE 22185 Lund, Sweden; 3 Department of Pathology, Regional Laboratories Region Skåne, SE 22185 Lund, Sweden; 4 Department of Pathology, Karolinska University Hospital, Stockholm, Sweden; 5 Department of Pathology and cytology, Sahlgrenska university hospital, Gothenburg, Sweden; 6 Department of Respiratory Medicine, Sahlgrenska University Hospital, Gothenburg, Sweden; 7 Department of Pulmonary Medicine, University hospital Linköping, Linköping, Sweden; 8 Department of Pathology and Department of Clinical and Experimental medicine, Linköping University, Linköping, Sweden; 9 Respiratory Medicine Unit, Department of Medicine Solna and CMM, Karolinska Institutet and Karolinska University Hospital Solna, Stockholm, Sweden; 10 Centre for Research and Development, Uppsala University/Region Gävleborg, Gävle, Sweden; 11 Thoracic Oncology Unit, Karolinska University Hospital and Department Oncology-Pathology, Karolinska Institutet, Stockholm, Sweden; 12 Department of Immunology, Genetics and Pathology, Uppsala University, SE 75185 Uppsala, Sweden; 13 Department of Pathology, Umeå University Hospital, SE 90185 Umeå, Sweden; 14 Department of Public Health and Clinical Medicine, Division of Medicine, Umeå University, SE 90185 Umeå, Sweden; 15 Department of Radiation Sciences, Oncology, Umeå University, SE 90185 Umeå, Sweden; 16 Department of Respiratory Medicine and Allergology, Skåne University Hospital, SE, 22185 Lund, Sweden

## Abstract

Accurate histological classification and identification of fusion genes represent two cornerstones of clinical diagnostics in non-small cell lung cancer (NSCLC). Here, we present a NanoString gene expression platform and a novel platform-independent, single sample predictor (SSP) of NSCLC histology for combined, simultaneous, histological classification and fusion gene detection in minimal formalin fixed paraffin embedded (FFPE) tissue. The SSP was developed in 68 NSCLC tumors of adenocarcinoma (AC), squamous cell carcinoma (SqCC) and large-cell neuroendocrine carcinoma (LCNEC) histology, based on NanoString expression of 11 (*CHGA*, *SYP*, *CD56*, *SFTPG*, *NAPSA*, *TTF-1*, *TP73L*, *KRT6A*, *KRT5*, *KRT40*, *KRT16*) relevant genes for IHC-based NSCLC histology classification. The SSP was combined with a gene fusion detection module (analyzing *ALK*, *RET*, *ROS1*, *MET*, *NRG1*, and *NTRK1*) into a multicomponent NanoString assay. The histological SSP was validated in six cohorts varying in size (n = 11–199), tissue origin (early or advanced disease), histological composition (including undifferentiated cancer), and gene expression platform. Fusion gene detection revealed five *EML4-ALK* fusions, four *KIF5B-RET* fusions, two *CD74-NRG1* fusion and three *MET* exon 14 skipping events among 131 tested cases. The histological SSP was successfully trained and tested in the development cohort (mean AUC = 0.96 in iterated test sets). The SSP proved successful in predicting histology of NSCLC tumors of well-defined subgroups and difficult undifferentiated morphology irrespective of gene expression data platform. Discrepancies between gene expression prediction and histologic diagnosis included cases with mixed histologies, true large cell carcinomas, or poorly differentiated adenocarcinomas with mucin expression. In summary, we present a proof-of-concept multicomponent assay for parallel histological classification and multiplexed fusion gene detection in archival tissue, including a novel platform-independent histological SSP classifier. The assay and SSP could serve as a promising complement in the routine evaluation of diagnostic lung cancer biopsies.

## Introduction

Lung cancer accounts for more than 1.6 million deaths annually worldwide, making it the deadliest form of cancer^1^. Non-small cell lung cancer (NSCLC) is the predominant subtype, which is further divided based on histological growth pattern. The two major NSCLC histological subtypes are adenocarcinoma (AC) and squamous cell carcinoma (SqCC)^2^. Large-cell lung carcinoma (LCC) and large-cell neuroendocrine carcinoma (LCNEC) are less common but represent important differential diagnoses. Histological assessment of lung cancer is clinically important, since histological subtype can affect clinical management regarding, e.g., choice of therapy^3–5^. Standard diagnostic procedures in histological subtyping include assessment of microscopic morphology and immunohistochemical (IHC) analysis of protein marker expression. The WHO guidelines from 2015 suggest to classify poorly differentiated tumors that express either TTF-1 or napsin A (NAPSA) as AC, while tumors that express either CK5 (keratin 5, *KRT5*) or p40 are classified as SqCC^2^. However, in advanced NSCLC disease a substantial proportion of tumors (approx. 20%)^6,7^ are for various reasons not eligible for subtyping. These tumors are referred to as NSCLC - not otherwise specified (NSCLC-NOS), and have been associated with worse outcome in advanced disease^8^. A tool for histological delineation of this patient group may lead to improved clinical management.

Besides histological subtype, clinical routine diagnostics of lung cancer today involves assessment of a number of key treatment predictive molecular alterations, including both activating mutations (e.g. in *EGFR*) and various gene fusions. In 2007, Soda and colleagues reported the *EML4-ALK* fusion as a potentially new molecular and therapeutic marker in lung cancer^9^. Over the years, additional gene fusions have been mapped and shown to have therapeutic value in lung cancer. Consequently, gene fusion analyses of *ALK* and *ROS1* are now routine diagnostic practice for the majority of advanced stage patients based on IHC and/or fluorescence *in-situ* hybridization (FISH). Additional fusion genes are likely to be included for screening in the near future. As clinical lung cancer specimens (tissue) from advanced stage patients are often scarce due to small biopsies and the number of treatment predictive genes to test increases, a combined multicomponent assay for parallel histological assessment, fusion gene detection and mutation screening would be preferred to save time and tissue, and reduce cost. Along this line, next-generation sequencing (NGS) for clinical diagnostics are rapidly becoming general diagnostic practice, providing information on a variety of treatment predictive gene mutations, and for specific NGS panels also genes fusions based on dual analysis of DNA and RNA extracted from the same tumor specimen. While the latter type of panels can provide data on specific fusions they do have limitations^10^. Firstly, RNA is needed for library preparation, which may be challenging considering the degradation that occurs during fixation and storage. Secondly, regional clinical laboratories are often limited to the use of focused NGS panels (i.e., analyzing a smaller set of genes on the DNA level only) as whole genome sequencing, whole exome sequencing, or sequencing of larger targeted panels (like combined DNA and RNA panels) remains cost- and time consuming, demanding regarding sample throughput, challenging concerning archival tissue, and generates more information than manageable (and needed) in day-to-day practice.

As an alternative to NGS-based RNA fusion gene analysis, the NanoString technology, an RNA based technology based on capture of targets specified by the user, enables focused gene expression profiling and/or multiplexed gene fusion detection in one assay from small amounts of degraded RNA. This open platform thus allows for the creation of RNA-based multicomponent assays addressing different clinical needs, simultaneously saving time, tissue, and cost. For instance, a single RNA-based assay can be imagined that combine gene fusion detection of multiple genes, histological subtyping, and novel treatment predictive or prognostic signatures based on analysis of expression of different gene sets. The NanoString method is the basis for the ProSigna® assay, which is a clinical assay for chemotherapy treatment decision making in breast cancer using formalin-fixed paraffin embedded (FFPE) tissue (www.nanostring.com). The assay is used in a decentralized manner at regional pathology departments in several countries worldwide.

In this study, we aimed to test the novel concept of a multicomponent RNA-based diagnostic tool for parallel histological subtype prediction (AC, SqCC, and LCNEC) (primary aim) and gene fusion analysis (secondary aim) suitable for archival lung cancer tissue, representing two independent aims addressed by a single assay. Thus, the proposed tool would perform two of three routine clinical tasks (gene fusion analysis and histological classification, but not mutational analysis). This task required development of a combined experimental assay (suitable for both aims) and a novel prediction algorithm (for the histology aim). As the experimental basis we used an existing, validated, NanoString assay for detection of gene fusions in clinical NSCLC tissue^11^. This assay was extended with additional fusion genes and again validated in response to the secondary aim of RNA-based fusion gene detection. To this new extended assay we also added key prototypic lineage-related genes (n = 11) of which many are currently used as clinical IHC markers for histological subtyping of NSCLC to also, simultaneously, be able to achieve the primary aim of histological subtyping. The reasons for selecting this limited key set of genes were to: (1) facilitate interpretation of results, (2) mimic the usage of prototypic lineage genes as in IHC, (3) limit the size (number of genes) of a final assay to reduce cost, and (4) allow greater cross platform potential as such key genes are likely to be either included, or more easily included, in assays from other platforms (like NGS platforms). The main challenge in this study consisted of deriving a novel type of predictor for histological subtype using the expression of the diagnostic genes. For clinical applicability, and in contrast to other studies deriving gene expression predictors of lung cancer histology^12–15^, we aimed to derive a classifier capable of classifying samples independent of other cases (a so-called single sample predictor, SSP) irrespective of gene expression platform. Thus, the derived predictor should in theory be platform-naïve, meaning that it could be applicable to data from, e.g., qPCR, NanoString, microarrays, or NGS platforms. Based on analyses in an FFPE training cohort we derived a subtype predictor with good performance in independent cohorts comprising both fresh frozen and archival tissue from early stage or advanced patients including undifferentiated cancers and different technology platforms according to the current guidelines for lung cancer classification (WHO 2015). Our results demonstrate that a multicomponent gene expression assay combining histological subtyping and gene fusion detection could be a useful complement in clinical diagnostics of lung cancer as previously reported^11^, and we in addition present a bioinformatical classifier useful for gene expression data derived from e.g. RNA sequencing.

## Materials and Methods

### Ethics statement

The study was approved by the Regional Ethical Review Board in Lund, Sweden (registration numbers: 2004/762, 2008/702, 2014/546, 2014/748, 2015/575 and 2015/831). By decision of the Ethical Review Board, specific written informed consent from included patients in this study were not required if these were not included in the ongoing LUCAS study (for which written informed consent existed), as no personal data was used for this study. In accordance with the decision of the Ethical Review Board, non-LUCAS patients were informed about the study through local advertisement in news media in the region. All experiments were conducted in agreement with patient consent and ethical review board regulations and decision.

### Patient material – Single Sample Predictor (SSP) development cohort

**Figure 1:**
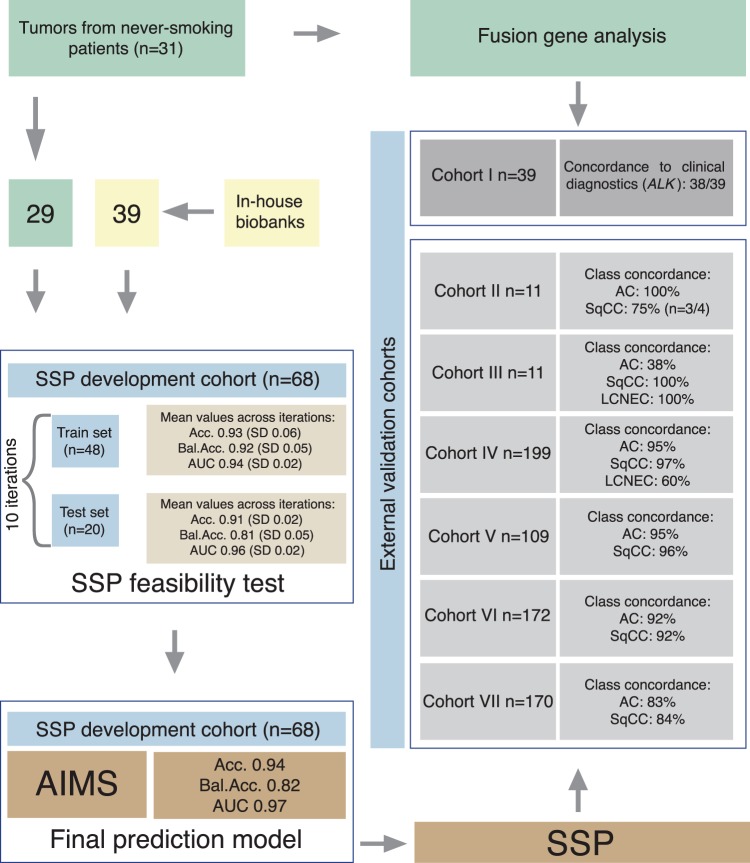
Cohorts and SSP development. 31 tumors from never-smokers were profiled by NanoString analysis. 29 of these patients and 39 tumors of SqCC and LCNEC histology from in-house biobanks were merged to a final SSP development cohort (n = 68). A feasibility test of the SSP was performed prior to deriving a final prediction model. In the feasibility test, samples were partitioned based on histology into a train and test set respectively and iterated 10 times. The SSP developed during the feasibility test in the train set was used to classify tumors of the test set and re-classify tumors of the train set. Accuracy, balanced accuracy and AUC values were calculated as mean values over iterations. Based on the high performance and low variability due to different sample selections of the iterated SSP models in the feasibility test, a final prediction model was trained in the entire SSP development cohort (n = 68) and used for re-classification of tumors in the SSP development cohort for confirmatory purpose. To test the independent performance of the final SSP, the model was applied to six external validation cohorts that differed in size, tumor stage, histology composition and gene expression data platform. Fusion gene detection using the NanoString assays was performed on a cohort of never-smoking patients (n = 31), the SSP development cohort, and three validation cohorts.

FFPE sections from 31 surgically resected tumors of never-smoking patients diagnosed 2005–2015 was obtained from Umeå University Hospital as part of a national multi-center study. 29 of these tumors were of AC (n = 27) or SqCC (n = 2) histology and selected for classifier development. Two cases were of other, rarer, histological subtypes and not included in SSP development. Additional tumors with SqCC (n = 28) and LCNEC (n = 11) histology were obtained from other studies^16,17^ to create the final SSP development cohort (n = 68) (Table 1, Fig. 1). All histological classifications were performed by pathologists prior to NanoString gene expression analyses.

**Table 1:** Patient cohorts and clinicopathological features.

Histological assessment by pathologist	Cohort								
	Fusion gene detection**	SSP development cohort	External validation cohort I***	External validation cohort II^◆^	External validation cohort III^◆^	External validation cohort IV^◆◆^	External validation cohort V^◆◆^	External validation cohort VI^◆◆^	External validation cohort VII^◆◆^
AC	78	27	39	4	8	115	83	106	127
SqCC	35	30	—	4	1	68	26	66	43
LCNEC	12	11	—	—	1	5	—	—	—
NSCLC-NOS	1	—	—	—	1	—	—	—	—
LCC	3	—	—	3	—	6	—	—	—
Other*	2	—	—	—	—	5	—	—	—
Total	131	68	39	11	11	199	109	172	170
Intended use of data	NanoString fusion gene detection ability	Fusion gene detection SSP training and testing	NanoString fusion gene detection ability	Fusion gene detection SSP validation	Fusion gene detection SSP validation	SSP validation	SSP validation	SSP validation	SSP validation
Data generation platform	NanoString	NanoString	NanoString	NanoString	NanoString	RNAseq	Illumina microarrays	Affymetrix microarrays	Affymetrix microarrays
Novelty data generation	Yes	Yes	Yes	Yes	Yes	No	No	No	No
Tissue origin	FFPE	FFPE	FFPE	FFPE	FFPE	Fresh frozen	Fresh frozen	Fresh frozen	Fresh frozen

*This subgroup includes sarcomatoid carcinomas, carcinoid tumors, and adenosquamous carcinomas.

**Fusion gene analysis was performed in all samples from the SSP development cohort, and validation cohort I, II and III plus two tumors of other histological subtypes.

***Patients with a never-smoking history and surgically resected tumors.

^◆^Advanced disease. Biopsies and cytologies.

^◆◆^Surgically resected tumor material.

### Patient material - External validation cohorts

Seven external validation cohorts were assembled. Cohort I contained 39 tumors of AC histology (FFPE tissue) from never-smoking patients with primary lung cancer enrolled in the same multi-center study as patients included in the SSP development cohort. The purpose of validation cohort I was to confirm the ability of the NanoString assay to detect clinically determined *ALK* gene fusions in an independent cohort. Clinical *ALK* gene fusion status was determined by IHC and/or FISH or qPCR performed according to clinical routine protocols at each site as part of the standard diagnostic routine. At the time of diagnosis for these samples no other fusion genes besides *ALK* were tested routinely. Cohort II comprised of 11 tumors originally classified as LCC by the WHO 2004 guidelines with updated classification according to WHO 2015 guidelines^16^. Cohort III comprised of 11 advanced stage NSCLC-NOS patients previously reported by Ericson-Lindquist *et al*.^11^. The 11 NSCLC-NOS cases were the maximum number of cases that could be retrieved with sufficient FFPE tissue left for research purposes after routine diagnostics. For this study, NSCLC-NOS cases were in-depth reviewed with complementing morphological assessments and diagnostic IHC analyses, including periodic acid–Schiff–diastase (PAS-D), NAPSA, TP73L, Ki-67, CD56, and synaptophysin (SYP) stains, to determine the histological subtype (Supplemental Table 1). Histopathological re-review was performed blinded from the SSP prediction analysis. Experimental data for cohorts I, II, and III were generated for this study using the same NanoString assay as the for SSP development cohort and all histological classifications were performed by pathologists prior to NanoString analyses. Cohort IV comprised of reported RNA sequencing (RNAseq) data from 199 analyzed surgically resected lung specimens described by Djureinovic *et al*.^18^, with histological subtypes updated according to WHO 2015 guidelines^2^. Cohorts V (GSE94601)^16^, VI (GSE37745)^19^ and VII (GSE50081)^20^ comprised of publicly available gene expression data generated using microarrays (Illumina HT-12 v4 in cohort V, Affymetrix Human Genome U133 Plus 2.0 Array in cohorts VI and VII). Cases of LCNEC histology in validation cohort V were excluded as most of these cases were included in the SSP development cohort. Due to an inconsistency of used WHO guidelines for histological classification, only cases with AC and SqCC histology were selected in VI (n = 172) and VII (n = 170). Cohorts are further described in Table 1.

### RNA extraction and quality control

RNA and DNA from FFPE sections were extracted using the Qiagen AllPrep DNA/RNA FFPE Kit (catalogue number 80234, Qiagen, Hilden, Germany). RNA was quantified using the NanoDrop (ThermoFisher Scientific, Waltham, MA, USA) and evaluated on the Bioanalyzer (Agilent Technologies, Santa Clara, CA, USA) using DV_200_ (percentage of fragments >200 nucleotide) as an indicator of RNA quality prior to further analysis.

### NanoString analysis

NanoString analysis, novelty generated, was performed on all samples in the SSP development cohort and validation cohorts I, II and III (Table 1, Supplemental Tables 2 and 5) using an RNA-based nCounter Elements assay (NanoString Technologies, Seattle, WA, USA). A probe set was designed based on prior analysis^11^ and a literature search^21–23^ (Supplemental Tables 3). In this updated probe set, new fusions were added along with probes corresponding to the IHC markers used to define histological subtypes in lung cancer, as well as immune markers and proliferative markers. NanoString analysis was performed on a Sprint instrument according to manufacturer’s instructions. Fusion positive controls were included and fusion gene prediction was performed analytically as previously described^11,24^.

### Single sample predictor development and validation

Background corrected raw counts corresponding to 11 genes (LCNEC: *CHGA*, *SYP*, *CD56*, AC: *SFTPG*, *NAPSA*, *TTF-1*, SqCC: *TP73L*, *KRT6A*, *KRT5*, *KRT40*, *KRT16*) used in clinical diagnostics or shown to form specific gene expression modules related to lung tumor histologies^25^ were extracted from the NanoString data. For the publicly available validation cohort IV (GSE81089)^18^, we used uncorrected Fragments Per Kilobase Million (FPKM) counts as validation cohort IV contains data generated using RNAseq. Non-normalized microarray data were used for validation cohorts V, VI and VII. For the development cohort (consisting of newly generated NanoString data), we first partitioned the cohort into a training (n = 48) and test cohort (n = 20) balanced for tumor histology (AC, LCNEC, SqCC) using the Caret R-package and conducted a predictor feasibility test. A single sample predictor (SSP) was built using the Absolute Intrinsic Molecular Subtyping (AIMS) model^26^ (available as scripts from the original authors’ GitHub account) in the training set and evaluated in the test set using accuracy, balanced accuracy and area under curve (AUC) as performance metrics. We iterated this process 10 times to assure that sample partitioning did not greatly influence results, thus creating 10 different models with 10 respective metric values (accuracy, balanced accuracy, and AUC), using a mean value over the iterations for final SSP performance evaluation. The final SSP prediction model used in independent cohorts was created using the entire development cohort (n = 68) for training (Fig. 1). The SSP model and exemplary datasets for NSCLC histology prediction is available as an R package (SSP_NSCLC_histology.zip).

## Results

### Fusion gene detection using NanoString

**Figure 2:**
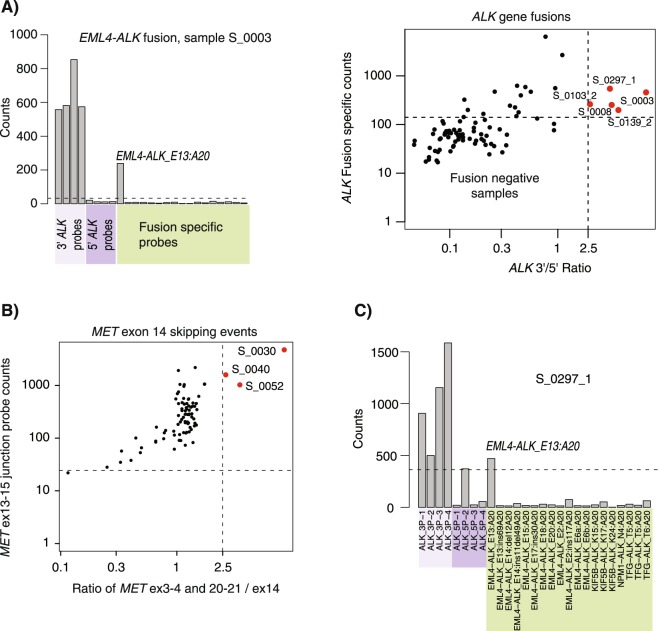
Detection of *ALK* fusion gene and *MET* exon 14 skipping events using the NanoString technology. (**A**) Detection of gene fusions, e.g. involving *ALK*, by the NanoString assay is based on expression (counts) of the 3′, 5′ part of the gene, and fusion specific probes as described elsewhere (see e.g.^11^). The actual count values (left panel) for *ALK* related probes in sample S_0003 reveals the, likely, exact ALK fusion (*EML4-ALK_E13:A20*) and demonstrates the differential expression of the 3′ and 5′ probes of the *ALK* gene when a fusion occurs. Combining a 3′/5′ probe ratio with fusion specific probe expression identifies five *ALK* fusion positive samples (red samples) in the upper right quadrant of a scatter plot of the 3′/5′ expression ratio versus the expression of *ALK* fusion specific probes (left panel) (see^11^ for further details) for cases subjected to fusion gene analysis (see Table 1). (**B**) Identification of three patients harboring *MET* exon 14 skipping events (red samples). Detection is based on high expression of a specific junction probe spanning exon 13–15 (excluding exon 14) (y-axis), versus a ratio of the mean expression for probes representing exons 3–4 and 20–21 divided by exon 14 specific expression (x-axis). Samples harboring *MET* exon 14 skipping events are visualized in the upper right quadrant as these report high junction probe counts and differential expression of exon 14 and exons 3–4 and 20–21 probe counts. (**C**) Raw NanoString count data for the S_0297_1 sample, which was tested clinically ALK positive by IHC, but was called FISH negative. NanoString analysis identifies a likely EML4-ALK_E13:A20 fusion. One 5′ probe demonstrates a high background count compared to remaining 5′ probes.

Fusion gene detection in all NanoString analyzed cases (development + validation cohorts I-III, see Table 1) revealed five *EML4-ALK* fusions (EML4-ALK_E6B:A20 (n = 1), EML4-ALK_E13:A20 (n = 3), EML4-ALK_E20:A20 (n = 1)) (Fig. 2A), four *KIF5B-RET* fusions (KIF5B-RET_K15:R12 (n = 2), KIF5B-RET_K16:R12 (n = 2)), two *CD74-NRG1* fusions (CD74-NRG1_C8:N6) and three *MET* exon 14 skipping events (Fig. 2B). All fusion positive and *MET* exon 14 skipping cases were found in the development cohort (which included two cases not used in later SSP development due to different histology) and validation cohort I (comprising of never-smokers). The high gene fusion frequency in the SSP development cohort is due to the inclusion of 31 never-smoking patients of primarily the AC histological subtype (7/31 = 23% fusion frequency in this subgroup alone). To validate the NanoString gene fusion detection versus clinical ALK status we compared NanoString derived ALK status versus clinically determined ALK status by IHC, FISH or qPCR in validation cohort I (Table 1). In this cohort, the NanoString assay identified three *ALK* fusion positive cases. Perfect agreement was observed in 38 of 39 cases (accuracy = 0.97, sensitivity = 1.0, specificity = 0.67) (Table 2). The one discordant case was found to be NanoString (EML4-ALK_E13:A20) and IHC ALK positive, but ALK FISH negative (Fig. 2C). That no fusions were detected in Validation cohorts II and III is not surprising considering their LCC and NSCLC-NOS status, features commonly not associated with occurrence of gene fusions^11,16,17^. Taken together, these results corroborate previous studies of the feasibility of a NanoString based gene fusion assay based on analysis of archival tissue^11,24^.

**Table 2:** Concordance of *ALK* gene fusion detection using the NanoString technology to the clinical diagnostic routine analysis (external validation cohort I).

	*ALK* fusion negative using the NanoString assay (n)	*ALK* fusion positive using the NanoString assay (n)
*ALK* fusion negative in the clinical diagnostic routine (n)*	36	1**
*ALK* fusion positive in the clinical diagnostic routine (n)*	0	2

*Methods used include IHC, FISH and/or qPCR.

**FISH negative, IHC positive.

### Single sample prediction - feasibility

To investigate the feasibility of an RNA-based (from archival tissue) histological subtype SSP, we first performed an iterative training-test machine-learning procedure in the development cohort using the pre-specified 11 genes (Fig. 1). Across 10 iterations, the mean accuracy for the training set was 0.93 (standard deviation (SD) = 0.06), and 0.91 (SD = 0.02) for the test set. Mean balanced accuracy for the training set was 0.92 (SD = 0.05) and 0.81 (SD = 0.05) for the test set. Mean AUC for the training set was 0.94 (SD = 0.02) and 0.96 (SD = 0.02) for the test set. These results demonstrate the potential to derive a high-performance predictor for lung cancer histological subtype based on a small number of gene rules applicable to RNA from archival tissue without the need of any data preprocessing.

### SSP – final prediction model

Based on the high performance and low variability of the iterated SSP models in the feasibility test, we hypothesized that an AIMS model trained on the entire development cohort would be the most appropriate way forward to: (1) avoid selecting a potentially overfitted model from the iterated approach, and (2) to increase the training data size. Consequently, we trained a final AIMS model using the entire SSP development cohort (n = 68), optimized to using 15 rules for prediction (Supplemental Table 4). Reclassification of the SSP development cohort resulted in high performance metrics (accuracy = 0.94, balanced accuracy = 0.82 and AUC = 0.97), as expected due to the circular nature of this analysis. To assess the true potential of the final SSP model for histological classification of NSCLC tumors we next applied it to six independent data sets (validation cohorts II-VII), with different patient numbers, histological composition, tumor differentiation, tissue origin (early or advanced disease), and gene expression platforms (Fig. 1, Table 3).

**Table 3:** Cohorts and SSP concordance.

Cohort	Fusion gene detection (n)	Fusion positive cases (n) (incl. *MET* ex 14 skipping)	SSP development (n)	AIMS prediction (n)	AC concordance (%)	SqCC concordance (%)	LCNEC concordance (%)	Discordance
Never-smokers	31	7 (22.6% frequency)	29	29	93% (n = 25/27)	90% (n = 27/30)	100% (n = 11/11)	—
In-house biobanks	39	0	39	39				
Validation cohort I	39	7	—	—	—	—	—	See Table 2
Validation cohort II	11	0	—	11	100% (n = 4)	75% (n = 3/4)	—	SqCC: Low p40 staining, basaloid
Validation cohort III	11	0	—	11	38% (n = 3/8)	100% (n = 1)	100% (n = 1)	AC: Low RNA quality (n = 2), poor differentiation/lack of mucin markers (n = 2), SqCC metaplasia (n = 1)
Validation cohort IV	—	—	—	199	95% (n = 109/115)	97% (n = 66/68)	60% (3/5) 100% after reanalysis	AC: Ends up in AC-enriched clusters SqCC: Ends up in SqCC-enriched clusters LCNEC (n = 2): RNA extracted from AC-component alone
Validation cohort V	—	—	—	109	95% (n = 79/83)	96% (n = 25/26)	—	
Validation cohort VI	—	—	—	172	92% (n = 97/106)	92% (n = 61/66)	—	
Validation cohort VII	—	—	—	170	83% (n = 106/127)	84% (n = 36/43)	—	

### Validation of the histological SSP in limited stage NSCLC with undifferentiated growth pattern

Validation cohort II (n = 11) consisted of NanoString derived data generated from fresh frozen tissue from surgically treated patients. These tumors were undifferentiated and classified as LCC by WHO 2004 guidelines and revised to AC (n = 4), SqCC (n = 4) or LCC (n = 3) by WHO 2015 guidelines mainly based on IHC staining results. The four former LCC tumors re-classified as AC (according to WHO 2015 guidelines) were also classified as AC by the SSP. Three of four (75%) of SqCC re-classified tumors were also classified as SqCC by the SSP, and one as LCNEC. The discrepant SqCC case was of the basaloid subtype and had no P40 protein expression. Thus, the SSP classified 7 out of 8 AC and SqCC (88%) correctly (Table 3). For the three LCC cases, two were classified as LCNEC and one as SqCC by the SSP. It should be noted that the SSP was not trained to identify WHO 2015 LCC cases (which should not express any diagnostic marker genes), and that the misclassification into especially LCNEC may be adjusted by analysis of expression of prototypic LCNEC associated genes (expected to be significantly elevated in LCNEC). Overall, LCC cases reported low gene expression counts for analyzed genes supporting the previously noted “marker null” phenotype of these tumors^16^.

### Validation of the histological SSP in advanced stage NSCLC with undifferentiated growth pattern

**Figure 3:**
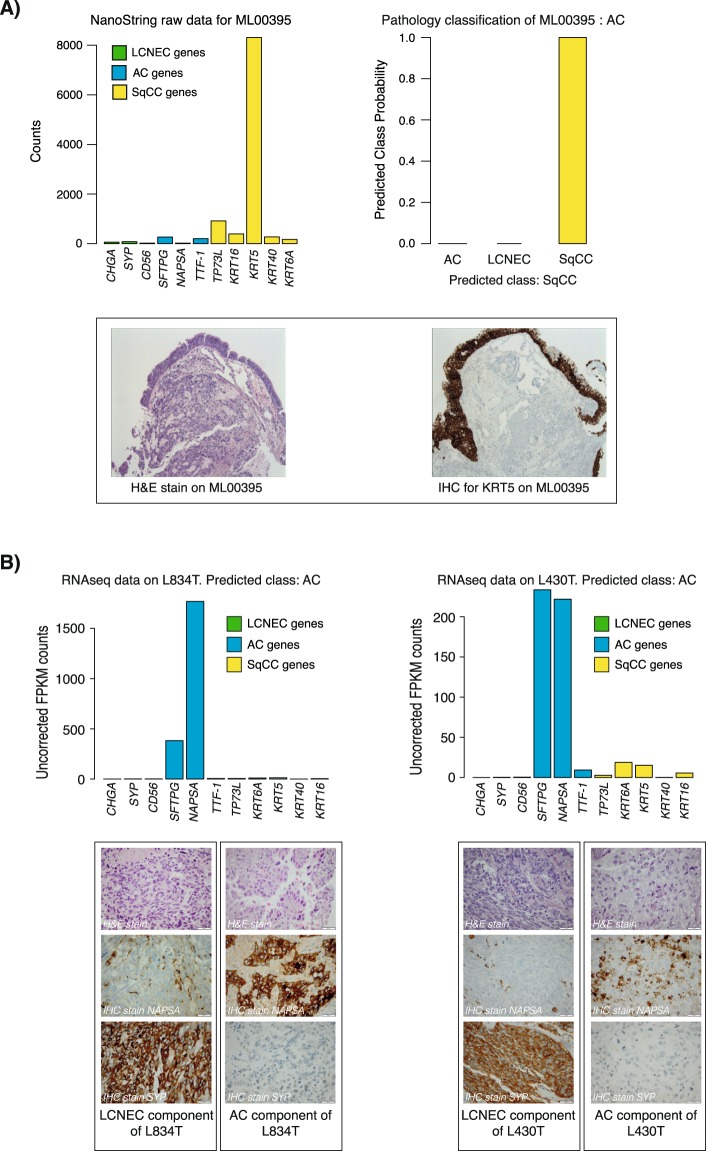
Discordance in SSP classification. (**A**) In-depth histopathological evaluation of a tumor initially classified as NSCLC-NOS that was re-classified as AC by a pathologist but SqCC by the SSP (top right panel) in validation cohort III. Hematoxylin staining of the tumor clearly demonstrates the tumor cell rich area (lower left IHC panel), while KRT5 IHC staining reveals positivity in bronchial epithelium with squamous metaplasia surrounding the tumor tissue (lower right IHC panel). The latter is most probably the cause of elevated *KRT5* (and other squamous markers) expression in the RNA extracted from the tissue (top left expression panel). (**B**) Two misclassified LCNEC tumors in validation cohort IV. Due to low mRNA expression of LCNEC associated genes and high expression of AC genes, these tumors are classified as AC by the SSP (top expression panels), discordant to original histopathological assessment. In-depth review of these discordant cases revealed mixed AC and LCNEC histology, evident from IHC stains using AC and LCNEC immunomarkers respectively (lower panels). Further review of these cases revealed that RNA had been extracted and analyzed by RNAseq only from the AC component from these two tumors.

Validation cohort III (n = 11) consisted of NanoString derived data from archival tissue from patients with advanced disease with tumors classified as NSCLC-NOS from routine clinical diagnostics^11^. All NSCLC-NOS cases were re-classified as AC (n = 8), SqCC (n = 1), NE (n = 1) or NSCLC-NOS (marker null, n = 1) by a pathologist (K.E-L). Concordance between histopathological class and SSP classification of SqCC and LCNEC tumors were 100% (Table 3). Three of eight (38%) re-classified AC tumors were also classified as AC by the SSP, while discordant AC cases were classified as SqCC by the SSP (Table 3). All discordant cases were reclassified as AC based on one or two positive mucin markers (CDX2 or PAS-D respectively, Supplemental Table 1). Tumors with this staining pattern are commonly associated with poor differentiation requiring special attention in subtyping^2,27,28^. Case-to-case review of the NanoString results from the five discordant AC tumors revealed overall low gene expression in two cases, likely due to low RNA quality (i.e., representing inconclusive analyses). One tumor presented with high expression of different keratins, e.g. keratin 5 (*KRT5*, referred to as CK5 in IHC) in the NanoString analysis, shifting the SSP classification towards SqCC. The high gene expression of *KRT5* was presumably due to expression in the normal bronchial epithelium including columnar and basal cells and squamous metaplasia surrounding the tumor, which was evident when reviewing hematoxylin and KRT5 (CK5) IHC stains (Fig. 3A), as these areas were included in the whole sections taken from the tissue block for RNA extraction. The remaining two discordant, poorly differentiated, cases were histopathologically classified as AC based on expression of mucin markers alone, for which no NanoString probe existed.

### Validation of the histological single sample predictor in unselected limited stage NSCLC samples

Validation cohort IV (n = 199) consisted of RNAseq derived data generated from fresh frozen tissue of surgically treated patients with tumors classified as AC, SqCC, LCNEC, LCC, adenosquamous or sarcomatoid according to WHO 2015 guidelines. In cohort IV, 95% (n = 109 of 115) and 97% (n = 66 of 68) of the tumors with AC or SqCC histology, respectively, were classified correctly by the SSP, while the corresponding initial success rate for LCNEC tumors was 60% (three of five cases) (Table 3). Interestingly, the two misclassified LCNEC tumors by the SSP did not seem to express LCNEC marker genes (Fig. 3B). Notably, a recent study did also not classify these tumors as LCNEC, instead subgrouping these tumors in transcriptional subgroups dominated by AC cases^16^. In fact, a detailed histopathological re-review of these two cases revealed a mixed histological subtype of LCNEC and AC in both cases, with fresh tissue sampling and RNAseq analysis only from the AC component (Fig. 3B). Together, this explains our predictor’s discrepant classification versus the originally reported histopathological classification, meaning that the SSP reached 100% concordance with current LCNEC histology in validation cohort IV. Overall accuracy for histopathologically classified AC, SqCC, LCNEC cases in validation cohort IV was 0.95 not accounting for the two LCNEC cases with sampling bias, and 0.96 if treating these cases as AC. For the latter context, for individual histological classes, sensitivity and specificity were 0.95 and 0.97 for AC, 1 and 0.97 for LCNEC, and 0.97 and 0.99 for SqCC, respectively.

For remaining discordant cases (six AC and two SqCC of 188 AC, SqCC and LCNEC in total) we analyzed these in the context of global gene expression by unsupervised consensus clustering of ~3000 highly varying genes, as well as biological metagenes as described previously^16^ (Supplemental Fig. 1). This analysis identified discordant cases in transcriptional clusters dominated by the SSP predicted group (e.g. histopathological SqCC in transcriptional AC clusters), as well as a set of AC cases with high expression of genes associated with neurodevelopment and a LCNEC phenotype, suggestive of a neuroendocrine phenotype in these tumors (consensus cluster 6, Supplemental Fig. 1). The findings from the unsupervised clustering were further, independently, supported for discordant cases by expression of metagenes representing highly correlating gene sets associated with basal/squamous differentiation, napsin/surfactant expression, or neurodevelopment (Supplemental Fig. 1). In summary, this independent analysis supports that the majority of discordant cases in validation cohort IV, at least on a global transcriptional level, have a phenotype consistent with the 11-gene SSP classification.

To further validate the histological SSP’s accuracy, we validated the SSP in three additional validation cohorts (External validation cohorts V, VI and VII, Table 1) comprising of publicly available gene expression data generated using microarrays (n = 451, Illumina HT-12 v4 or Affymetrix Human Genome U133 Plus 2.0 Array). Only cases of AC and SqCC histology were predicted using the SSP due to a discrepancy in WHO guidelines used by pathologists for histological classification. High concordance rates (Table 3) were observed in cohorts V and VI (95% and 92% concordance rate for AC histology and 96% and 92% for SqCC histology, respectively), while a lower concordance rate was observed for validation cohort VII (83% for AC histology and 84% for SqCC histology). To investigate the lower concordance in validation cohort VII we plotted the gene expression of *Napsin A* and *KRT5* representing prototypical lineage like genes for AC and SqCC, respectively, versus sample groups defined by intersection of histopathological and SSP classes (Supplemental Fig. 2A,B). The expression of these genes clearly mimic the SSP prediction, demonstrating for instance that histopathological AC cases predicted as SqCC by the SSP has *Napsin A* and *KRT5* expression similar to cases predicted as SqCC by both predictors. Moreover, unsupervised clustering of the 170 cases in validation cohort VII using the 3000 most varying genes demonstrates that discordant cases cluster well in line with the SSP prediction (Supplemental Fig. 2C).

## Discussion

In today’s clinical setting, screening for treatment predictive mutations and gene fusions in combination with histological classification of lung tumors are important factors in the clinical management of lung cancer patients. Since tissue material in especially advanced disease is often limited due to small biopsies and multiple routine analyses, a multicomponent assay handling several types of clinical tests is desirable from many aspects. In this study, we set out to test the novel concept of establishing a multipurpose assay for histological classification and parallel gene fusion detection (as described by us and others)^11,24^ based on analysis of gene expression patterns in archival tissue. We present both a single sample bioinformatical prediction algorithm for NSCLC tumor histology built around key diagnostic genes seemingly independent of gene expression platform, and a complete experimental multicomponent assay built on the NanoString platform for simultaneous histology assessment and complementary gene fusion detection applicable to archival tissue.

Regarding the secondary aim of the multicomponent assay, fusion gene detection, we have previously reported on the success of the NanoString technology to deliver fusion gene status for *ALK*, *RET*, and *ROS1* in a clinical setting based on analysis of 135 prospectively collected cases^11^. Here, we expand the previous validation of accurate *ALK* gene fusion detection by another 39 clinically tested cases (validation cohort I), demonstrating again the accuracy of the NanoString assay (Table 2), and that it may serve as an orthogonal method to resolve cases with discrepant IHC and FISH status. In addition, in the 36 cases tested clinically negative for *ALK* fusions in validation cohort I we detected three fusions and one exon skipping event involving other therapeutically targetable genes. These observations further demonstrate the advantage of a multigene fusion assay in clinical diagnostic routine. Finally, an assay such as the NanoString based one should also be able to detect intra-tumor heterogeneity concerning fusion expression, i.e., expression of different fusion transcripts. Taken together, our results validates others and ours’ reports^11,24^ of using NanoString as a robust and sensitive assay for fusion gene analysis in clinical tissue. It also illustrates the (likely clinical) importance of broad gene fusion screening in patients with a never-smoking history.

**Figure 4:**
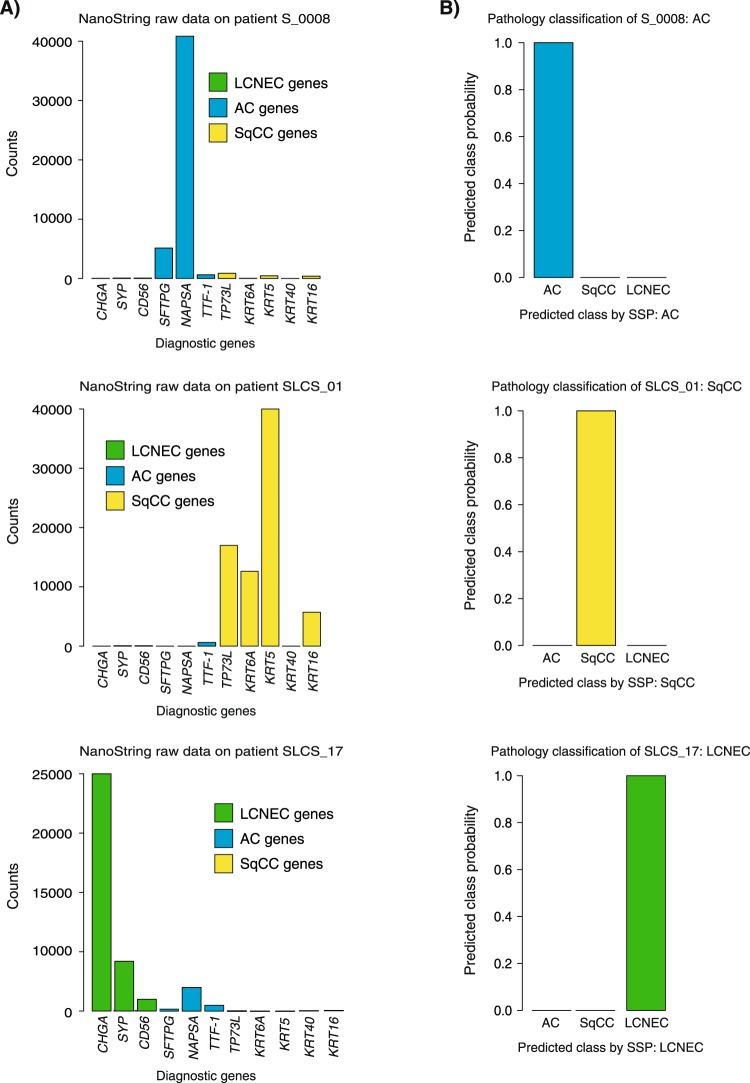
Examples of SSP classification. (**A**) SSP classification of three individual tumors corresponding to three major subtypes of NSCLC is based on expression of genes associated with AC histology (*SFTPG*, *TTF-1*, *NAPSA*), SqCC histology (*TP73L*, *KRT6A*, *KRT5*, *KRT40*, and *KRT16*) or LCNEC histology (*CHGA*, *SYP*, and *CD56*). (**B**) The SSP classifies these three individual tumors with high probability rates in concordance with the histopathological assessment of histology.

The primary objective of the multicomponent assay concerns gene expression driven prediction of tumor histology, representing the main focus of this study. Correct histological classification of NSCLC is clinically important, but may be challenging and time consuming due to technical issues (related to imaging and IHC staining), tissue source, tissue amount, poor tumor differentiation, and the large molecular heterogeneity of lung cancer. Addressing the primary objective implied both extension of the technical platform (NanoString) and development of a platform independent prediction algorithm. Importantly, the expression-based histological subtype predictor we aimed to derive should be able to independently classify single samples (i.e., an SSP) using only a limited set of genes, but also be applicable to different expression platforms to facilitate its use with other non-NanoString based clinical (e.g. RNA sequencing and microarrays) or research assays. Based on the AIMS machine-learning method^26^ (allowing >2 output classes which was needed for our study in contrast to other available binary SSP methods)^29,30^, we first performed a feasibility test showing that our simplistic approach of using only the expression of key diagnostic/lineage marker genes provided high classification performance for AC, SqCC and LCNEC prediction (Fig. 1). In this process, the skewness of the training cohort regarding proportions of histological subtypes (proportions of AC versus SqCC versus LCNEC), and never-smokers versus smokers is likely not an issue, as we search for prototypical (almost lineage-like) gene rules between histological subtypes. The latter may be exemplified by that same diagnostic IHC markers are used for diagnosis of both smokers and never-smokers. A final prediction model was applied to six external validation cohorts of different types (disease stage, histological subgroups, tumor differentiation, tissue origin), sizes, and analysis platforms in order to independently evaluate the SSP’s prediction performance in relevant and diagnostically challenging tumor subgroups. In spite of these fundamental differences, the derived histological SSP successfully classified the majority of samples histologically in line with the histopathology assessment (success rates per cohort is reported in Fig. 1 and Table 3 and illustrated for a set of individual cases in Fig. 4). Prediction of histology and concordance to histopathological assessments in validation cohort IV, a large cohort with a variety of histological subtypes analyzed by a gene expression platform (RNAseq) completely different from the SSP development cohort platform (NanoString), was strikingly accurate (95% concordance for AC and 97% for SqCC tumors). This demonstrates both the platform independency and accuracy of the predictor, irrespective of the patients smoking history (94% concordance for smokers with AC in validation cohort IV) despite the composition of the development cohort. The platform independency was further corroborated as the SSP proved highly accurate in prediction of AC and SqCC histology in validation cohorts V, VI and VII as these comprised of gene expression data generated using microarrays. In contrast to previously published predictors^12–15^, the SSP derived in this study is based on and applied to tumors histologically classified according to the WHO 2015 guidelines (validation cohorts IV and V), representing the golden standard in clinical routine today.

Reasons for observed discordance between SSP histology and pathologists’ classification differ between validation cohorts. The SSP was trained to identify the three most distinct subtypes of NSCLC: AC, SqCC and LCNEC based on a limited set of prototypic lineage genes. This methodological choice forces tumors of more well-defined, differing or mixed histological subtypes (e.g. sarcomatoid carcinomas, carcinoid tumors, LCC, or adenosquamous carcinomas) into one of the three classes leading to discordance. A striking example is LCC tumors in validation cohort II, which are defined by their lack of expression of AC/SqCC/LCNEC immunomarkers (“marker null”), but are still predicted by the SSP through the gene rule set-up of AIMS (as some expression is still noted for the 11 genes). It should also be noted that validation cohorts II and III are small in size (n = 11) leading to high discordance rates when merely one sample is misclassified. Validation cohort III comprise of challenging samples (NSCLC-NOS) due to poor differentiation, which require extra attention to be accurately classified through routine histopathological methods. We believe resolving such undifferentiated samples represent an important application type for any gene expression based assay. The challenging nature of NSCLC-NOS tumors is evident in the initially observed low concordance rate between the SSP and the histopathological re-review for AC cases in validation cohort III. However, discordant cases could be explained by either insufficient RNA quality (a challenge in archival tissue) or biological reasons such as SqCC metaplasia and diagnosis based on markers (mucins) not included in the current NanoString design. Importantly, these shortcomings can be addressed by: (1) an assay quality control step (see^11^), (2) appropriate micro/macro dissection considering the non *in situ* type of analysis, and (3) update of the NanoString probe content, respectively. Importantly, the discordance caused by insufficient RNA quality is an aspect limiting all types of RNA based clinical assays (including NGS based ones). Still, we do acknowledge that additional analyses of NSCLC-NOS cases are warranted to determine the optimal usage/design of the assay. We have demonstrated that the NanoString platform which the current study was built on achieved similar rates of conclusive *ALK* gene fusion analyses from FFPE RNA as IHC/FISH based analysis when tested in real clinical samples collected during one-year of routine analysis in a regional pathology department^11^. Notably, in validation cohort III, comprising of challenging FFPE-based NSCLC-NOS cases, we achieved a similar assay success rate as our previous study^11^ (~80%). While many assays claim to successfully analyze both mutations and fusions, the actual everyday success rate in regional laboratories may be different (and less successful) due to, e.g., tissue handling. Clearly, the usage of NanoString for decentralized clinical testing in breast cancer (the ProSigna® assay) implies that the technique is both robust and simple, which should be considered a strength for the multicomponent assay proposed in this study. In this context, we have also shown how NanoString based gene fusion detection may be incorporated in a clinical NGS-based framework for treatment predictive testing using amplicon-based NGS panels, with a two to three working day turn-around-time (excluding nucleic acid extraction) (see^11^).

In perspective of inter-observer rates reported between pathologists (77–95% for SqCC versus non-SqCC)^31–34^, the SSP appears highly accurate in predicting NSCLC histology (98.5% agreement for SqCC vs. non-SqCC in validation cohort IV). The SSP performed very well in the large WHO 2015 classified validation cohorts IV and V. In validation cohort IV, concordance rates for AC and SqCC were 95% and 97%, respectively, with an overall accuracy of 0.95 or 0.96 if correcting for the two LCNEC cases with discrepant tissue sampling, and with high group specific sensitivity and specificity (≥0.95). Discordant tumors were either of refined histology subtypes (adenosquamous, LCC, or sarcomatoid for which no SSP classes existed), or of AC/SqCC histology with expression of (discordant) AC, neuroendocrine or SqCC markers (Supplemental Fig. 1). The latter category of tumors (which have not been re-reviewed in this study) showed co-clustering with tumors of the corresponding SSP classification in independent unsupervised analysis. A similar finding was observed in validation cohort VII for discordant AC and SqCC cases (Supplemental Fig. 2C). Together, this suggests a potential of refined/revised classification by gene expression based methods (like the SSP), alternatively displays the difference of current *in situ* versus bulk tissue based classification (e.g. mixed tumor/microenvironment phenotype).

The gene expression based predictor may be evolved through several steps. These include: (1) addition of other well-defined histological subtypes (e.g. LCC, carcinoids etc.) to capture the full histological spectra of NSCLC in classifier training, (2) increase the number and diversity of NanoString probes to capture tumors of a histological subtype with a more specific expression pattern like poorly differentiated AC tumors expressing mucin markers like *CDX2* and *MUC* or *INSM1* (novel marker with higher specificity and sensitivity for LCNEC identification)^35^ and (3) train the predictor to find tumors of mixed histology by including also such tumors in the training. In addition, genes such as TS (thymidylate synthase) can be added to potentially support a pemetrexted treatment decision (high TS levels suggest less efficacy) – forming a new component of the assay related to treatment prediction.

In summary, we provide a first proof of concept of simultaneous fusion gene detection and histological classification using RNA from archival tissue through a package including both an experimental assay and a classification algorithm that may serve as a complementary diagnostic tool to e.g. NGS based mutational screening. Importantly, our derived histology classifier appears not restricted to NanoString data, but may be applied to other platforms as well given inclusion of the key diagnostic genes, including NGS-based ones that are currently the main workhorse in diagnostic laboratories. As even more information about the tumor is present in the RNA, additional modules can be added to the presented assay/concept, including, e.g., prognostic signatures or data on other (relevant) predictive markers (e.g. PDL1 and other immune infiltration markers). While our current assay does include such modules, they remain to be validated versus clinical predictions and patient outcome. The latter is especially interesting as archival tissue presents an option to macro/microdissect tissue to target relevant areas of a tumor, allowing bulk tissue analyses such as sequencing or transcriptional profiling to be interpreted in a more spatial, *in situ* like, context (while also potentially increase the accuracy of the analysis). In a disease characterized by high mortality rates and heterogeneous biology, accurate diagnostics exemplified by refined and efficient histological classification and treatment predictive tests are imperative for an improved, more stratified clinical management.

## Supplementary information

Supplemental Figures

Supplemental Tables

## Data Availability

All raw data generated using the described NanoString assay is available in the Supplemental Table file, together with training labels and predicted SSP classes.
