# Supplementary material for: A combined gene expression tool for parallel histological prediction and gene fusion detection in non-small cell lung cancer
Source: Sci Rep. 2019 Mar 26;9:5207. doi: 10.1038/s41598-019-41585-4 (PMC6435686; doi:10.1038/s41598-019-41585-4)
Supplement: Supplementary file 1 — Supplemental Figures [file 41598_2019_41585_MOESM1_ESM.pdf]

A combined gene expression tool for parallel histological prediction and gene fusion detection in non-small cell lung cancer

Anna Karlsson[1*], Helena Cirenajwis[1], Kajsa Ericson-Lindquist[2,3], Hans Brunnström[2,3], Christel Reuterswärd[1], Mats Jönsson[1], Cristian Ortiz-Villalón[4], Aziz Hussein[5], Bengt Bergman[6], Anders Vikström[7], Nastaran Monsef[8], Eva Branden[9,10], Hirsh Koyi[9,10], Luigi de Petris[11], Patrick Micke[12], Annika Patthey[13], Annelie F Behndig[14], Mikael Johansson[15], Maria Planck[1,16,A], Johan Staaf[1,A*]

[1] Division of Oncology and Pathology, Department of Clinical Sciences Lund, Lund University, Medicon Village, SE 22381 Lund, Sweden

[2] Division of Oncology and Pathology, Department of Clinical Sciences Lund, Lund University, SE 22185 Lund, Sweden

[3] Department of Pathology, Regional Laboratories Region Skåne, SE 22185 Lund, Sweden

[4] Department of Pathology, Karolinska University Hospital, Stockholm, Sweden

[5] Department of Pathology and cytology, Sahlgrenska university hospital, Gothenburg, Sweden

[6] Department of Respiratory Medicine, Sahlgrenska University Hospital, Gothenburg, Sweden

[7] Department of Pulmonary Medicine, University hospital Linköping, Linköping, Sweden

[8] Department of Pathology and Department of Clinical and Experimental medicine, Linköping University, Linköping, Sweden

[9] Respiratory Medicine Unit, Department of Medicine Solna and CMM, Karolinska Institutet and Karolinska University Hospital Solna, Stockholm, Sweden

[10] Centre for Research and Development, Uppsala University/Region Gävleborg, Gävle, Sweden

[11] Thoracic Oncology Unit, Karolinska University Hospital and Department Oncology-Pathology, Karolinska Institutet, Stockholm, Sweden

[12] Department of Immunology, Genetics and Pathology, Uppsala University, SE 75185 Uppsala, Sweden

[13] Department of Pathology, Umeå University Hospital, SE 90185 Umeå, Sweden

[14] Department of Public Health and Clinical Medicine, Division of Medicine, Umeå University, SE 90185 Umeå, Sweden

[15] Department of Radiation Sciences, Oncology, Umeå University, SE 90185 Umeå, Sweden.

[16] Department of Respiratory Medicine and Allergology, Skåne University Hospital, SE 22185 Lund, Sweden

*Corresponding authors

A: These authors contributed equally

A)

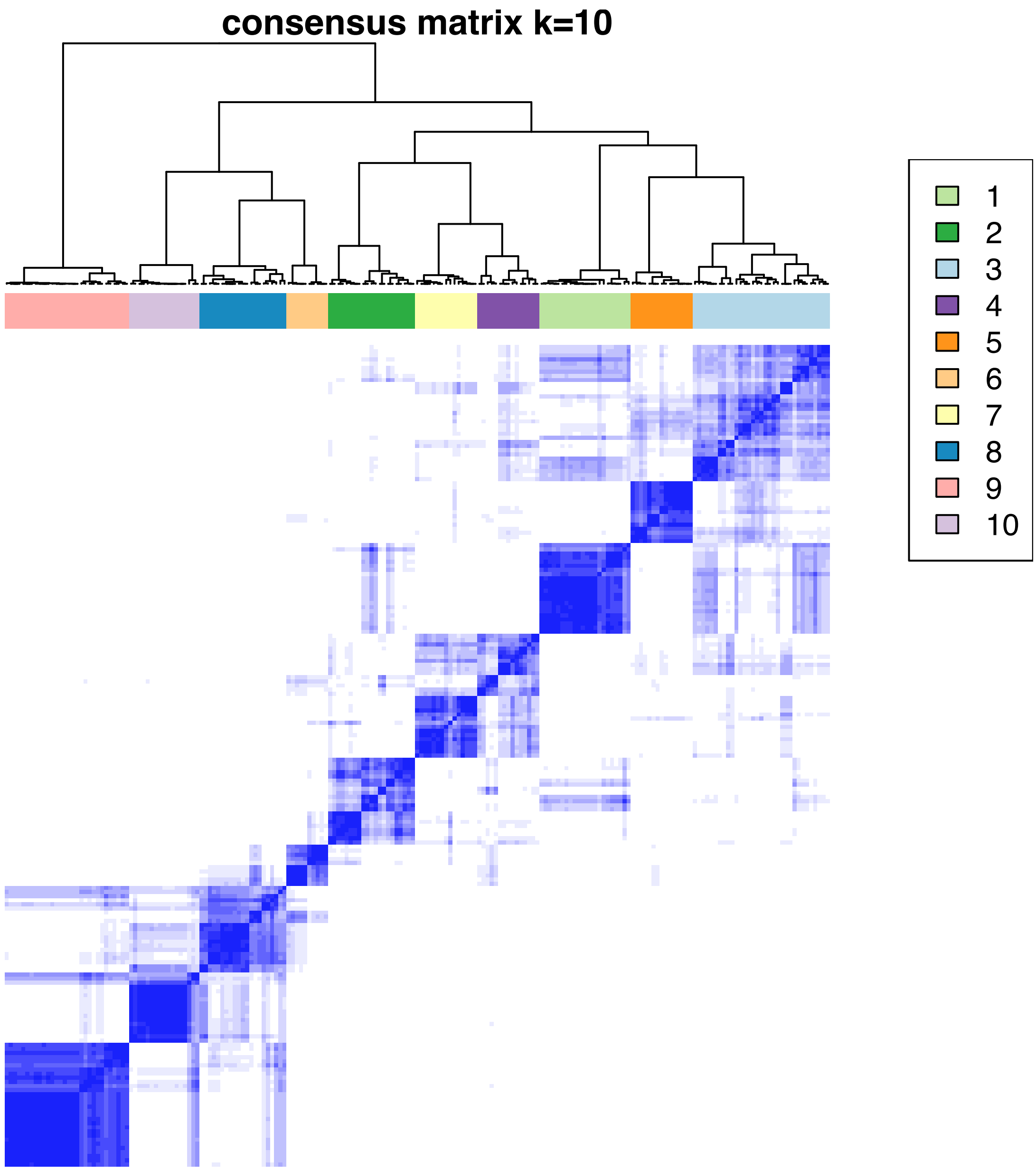

Supplemental Figure 1. Unsupervised clustering of validation cohort IV. Unsupervised consensus clustering was performed in validation cohort IV as described in Karlsson et al. JTO 2017. Briefly, FPKM data for 199 samples was log2 transformed after offset addition of +1 to all data, then mean-centered across all samples. Only genes with standard deviation >0.7 across all samples (representing genes with high variation across samples) were retained (n=3260). This data set was subjected to consensus clustering using the R-package ConsensusClusterPlus using 2000 iterations, pearson correlation as distant measure and ward's linkage. (**A**) Consensus clusters for a solution for 10 clusters showing that the identified clusters show high independence with cluster samples in clusters having high consensus (blue color, deeper color more consensus) showing low overlap with other clusters (white color) across all iterations and bootstraps. (**B**) Heatmap of log2 expression across the 199 cases ordered by their assigned consensus cluster (k=10). Arrows indicate cases with discrepant classification between pathology and the SSP. Annotation bars include expression of three different biological metagenes originally defined by Karlsson et al. CCR 2014. SARC: sarcomatoid. **(C)** Expression of a neurodevelopment metagene, a basal/squamous metagene, and a Napsin / surfactant metagene (originally from Karlsson et al. CCR 2014) versus combinations of pathological classifications and discrepant SSP classifications. Metagene scores were calculated as described in Karlsson et al. JTO, 2017. Top left panel illustrates expression of a neurodevelopment metagene for pathologically classified AC cases stratfied by SSP status, indicating high expression in three of five AC cases classified as LCNEC by the SSP. Top right panel shows expression of a basal / squamous metagene for pathologically classified AC cases and one case classified as SqCC by the SSP, demonstrating high expression in the latter suggesting a squamous phenotype or tissue context. Bottom two panels shows expression of the basal / squamous metagene and the Napsin / surfactant metagene for pathologically classified SqCC samples with two discrepant cases classified as AC by the SSP. The latter cases show low basal / squamous expression but high napsin / surfactant expression more consistent with an AC phenotype.

B)

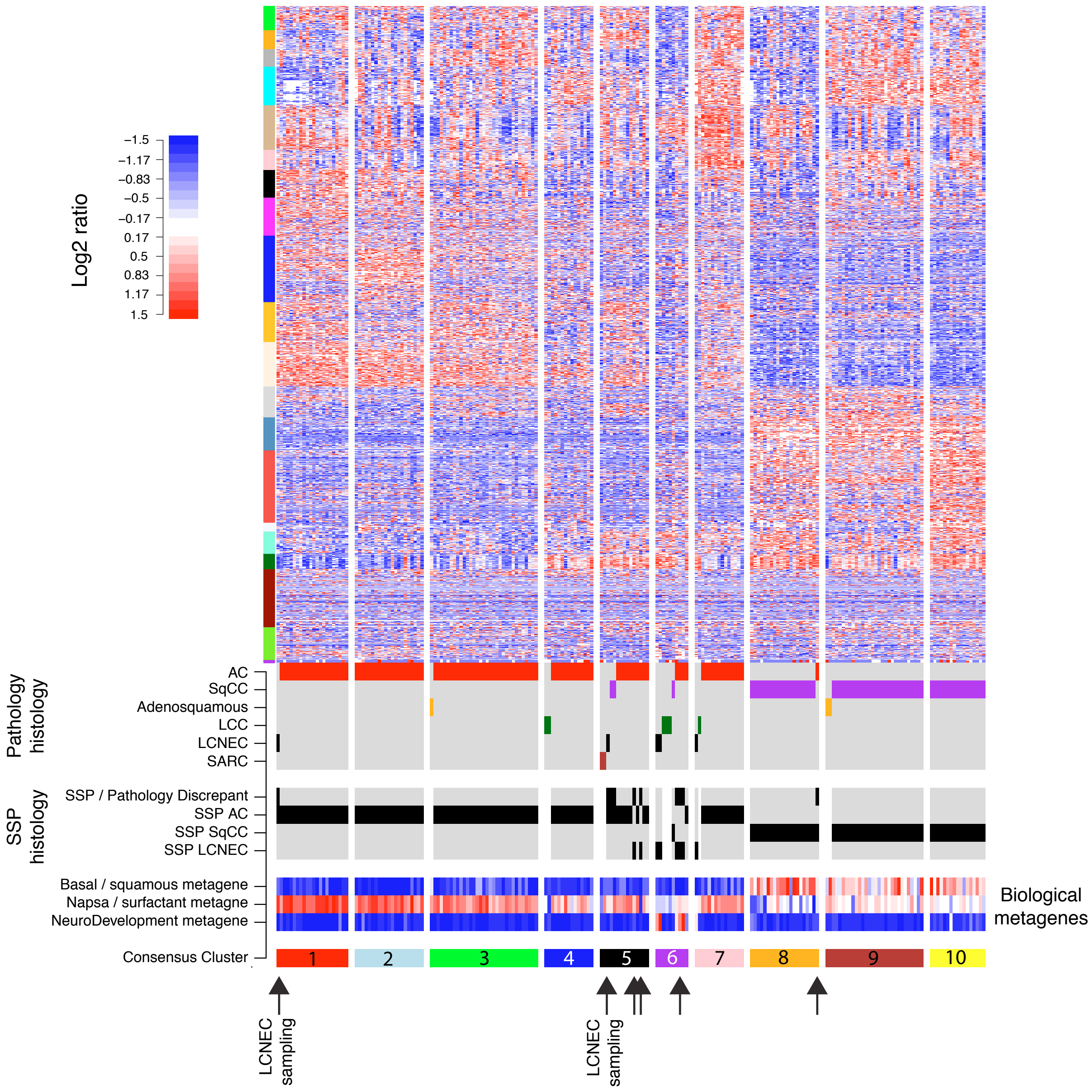

Log2 ratio
−1.5
−1.17
−0.83
−0.5
−0.17
0.17
0.5
0.83
1.17
1.5
Pathology histology
AC
SqCC
Adenosquamous
LCC
LCNEC
SARC
SSP histology
SSP / Pathology Discrepant
SSP AC
SSP SqCC
SSP LCNEC
Basal / squamous metagene
Napsa / surfactant metagne
NeuroDevelopment metagene
Biological metagenes
Consensus Cluster
1
2
3
4
5
6
7
8
9
10
LCNEC sampling
LCNEC sampling

C)

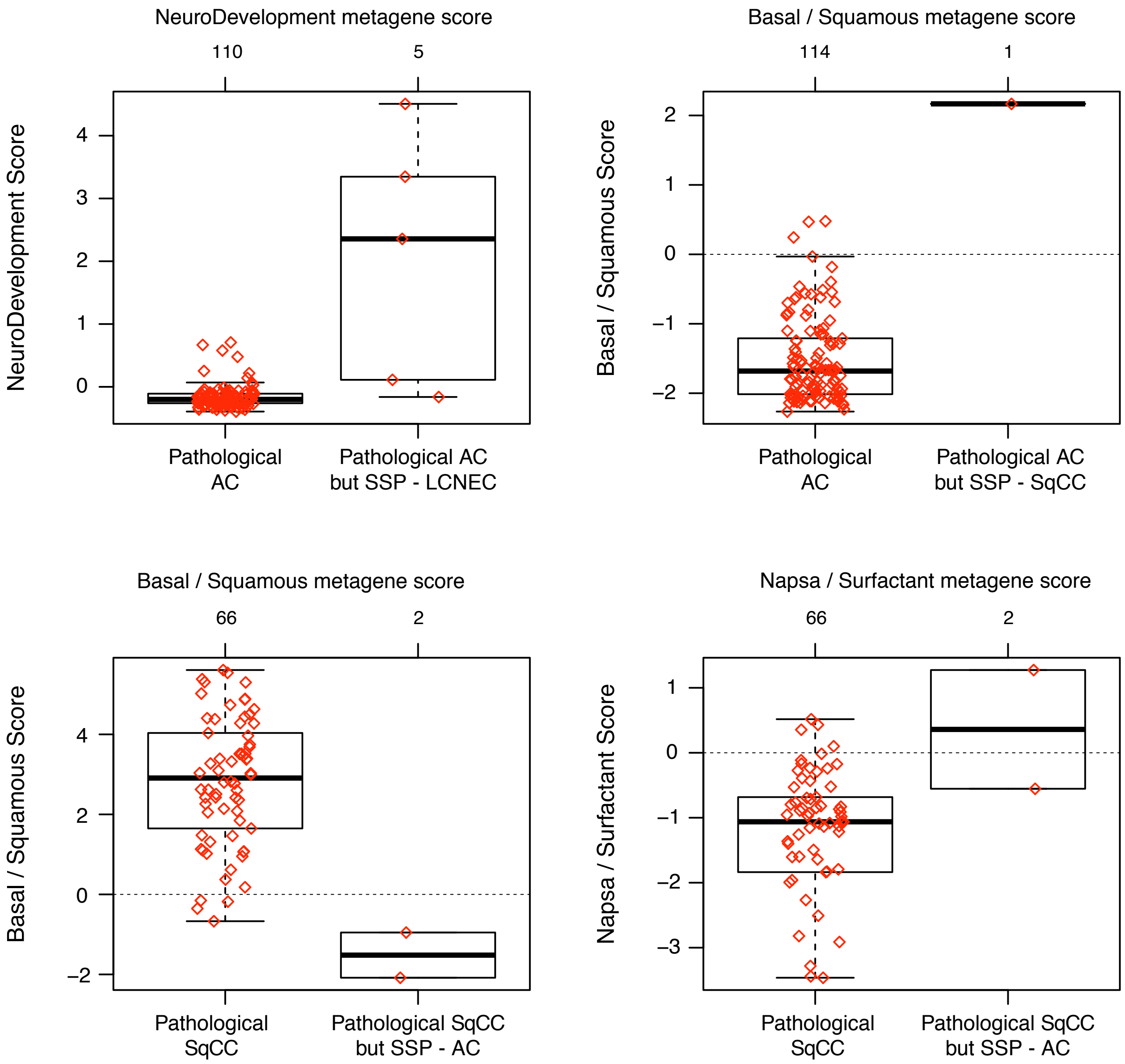
NeuroDevelopment metagene score
110
5
NeuroDevelopment Score
4
3
2
1
0
Pathological AC
Pathological AC but SSP - LCNEC
Basal / Squamous metagene score
114
1
Basal / Squamous Score
2
1
0
−1
−2
Pathological AC
Pathological AC but SSP - SqCC
Basal / Squamous metagene score
66
2
Basal / Squamous Score
4
2
0
−2
Pathological SqCC
Pathological SqCC but SSP - AC
Napsa / Surfactant metagene score
66
2
Napsa / Surfactant Score
1
0
−1
−2
−3
Pathological SqCC
Pathological SqCC but SSP - AC

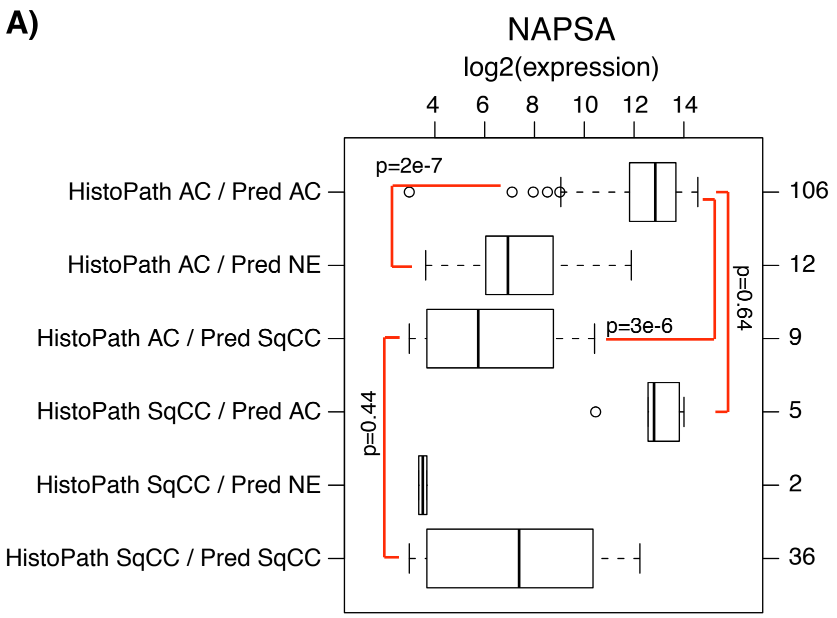

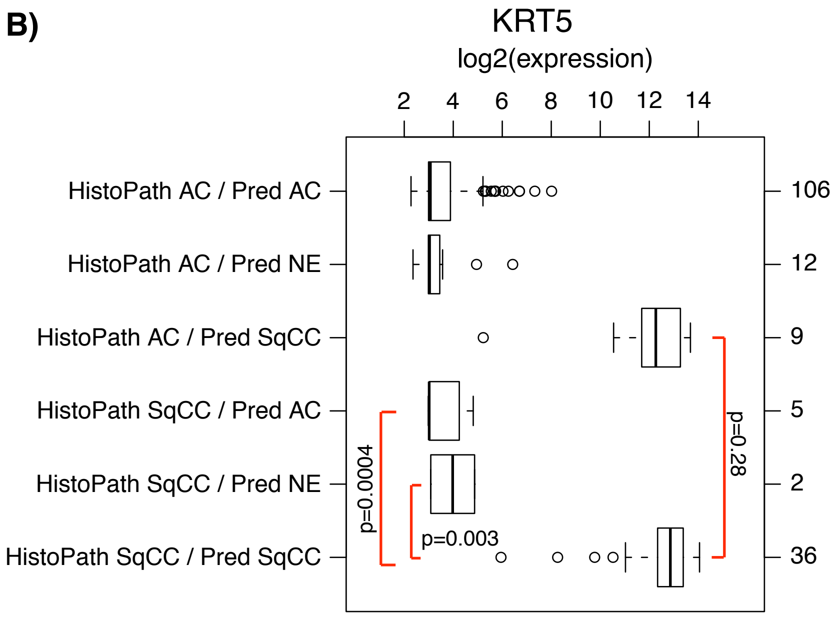

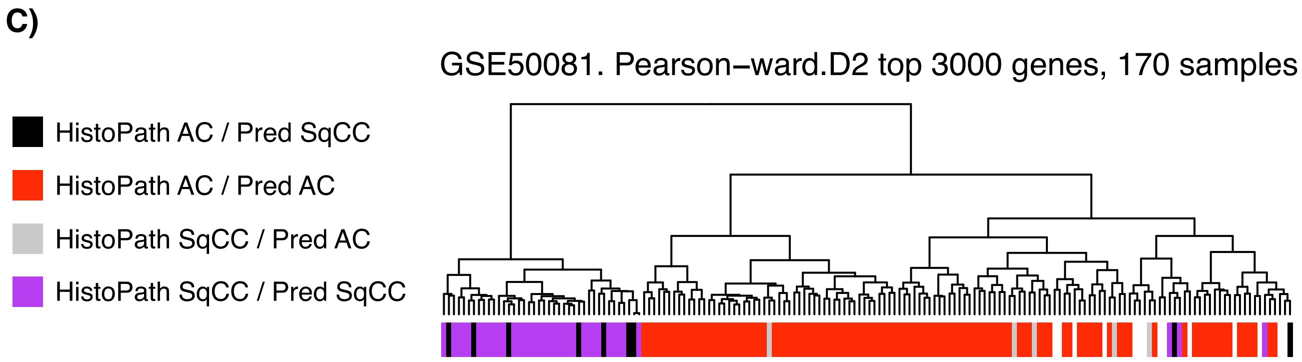

Supplemental Figure 2. *Napsin A* and *KRT5* gene expression in GSE50081 (validation cohort VII) stratified by histopathological and SSP prediction status. (**A**) *Napsin A* expression startified by histopathological tumor histology (HistoPath) and SSP histology (Pred). (**B**) *KRT5* expression startified by histopathological tumor histology and SSP histology. P-values calculated using Wilcoxon's test. All p-values reported are two-sided. Box-plot elements corresponds to: i) center line = median, ii) box limits = upper and lower quartiles, iii) whiskers = 1.5x interquartile range (**C**) Hiearchical clustering using Pearson correlation as distance metric and ward.D2 as linkage using the top 3000 most varying genes across the 170 samples in GSE50081. Sample dendrogram showed colored by histopathological and SSP prediction status with legend on the left.
